# Cognitive load, fatigue and aversive simulator symptoms but not manipulated zeitgebers affect duration perception in virtual reality

**DOI:** 10.1038/s41598-022-18520-1

**Published:** 2022-09-20

**Authors:** Magdalena Sabat, Bartosz Haładus, Michał Klincewicz, Grzegorz J. Nalepa

**Affiliations:** 1 Département d’études Cognitives, École Normale Superieure, PSL University, CNRS, 75005 Paris, France; 2 Department of Cognitive Science, Institute of Philosophy, Jagiellonian University, 31-007 Krakow, Poland; 3 Cognitive Science and Artificial Intelligence, Tilburg University, Werandelaan 2, 5037 AB Tilburg, The Netherlands; 4 Jagiellonian Human-Centered Artificial Intelligence Laboratory (JAHCAI) and Institute of Applied Computer Science, Jagiellonian University, 31-007 Krakow, Poland

**Keywords:** Human behaviour, Statistics

## Abstract

The perceived duration of an interval depends on numerous aspects of the passed event both endogenous, including physiological arousal, level of wakefulness, attention, and surprise, as well as exogenous such as valence, salience, or context in the environment. There is some evidence that "time-giving" cues from the environment (zeitgebers) are coupled with time perception. The movement of the sun on the horizon was demonstrated to affect interval perception in a study conducted by Schatzschneider et al. (2016) claiming that the sun’s motion is a zeitgeber that influences time perception. In the present study, we undertake the first to our knowledge replication of this effect, extending the analysis to confounding aspects of the used paradigm. We aimed to test the effect of immersion, cognitive load, and changes in the speed of the sun on the horizon of the virtual environment on the perceived interval duration. We did not replicate the original effect, as reported by Schatzschneider et al., however, we did find that the perceived duration of an interval was affected by cognitive load, fatigue, and unpleasant symptoms caused by VR. In our analysis, we used Bayesian statistics to support our conclusion and offer its results as having some important consequences for the field.

## Introduction

Time, understood as a feature of the environment, is a difficult concept that cannot be easily defined, but its interaction with cognitive and sensory processes has been studied extensively nonetheless^1^. From this work, we know that perception of time is unlike most other types of perception in that it does not have a dedicated receptor system or a single type of stimulus associated with it. Among many other factors, it can be affected by physical fatigue^2,3^, arousal^4–7^, emotional valence^4,8^, anxiety^9^, and cognitive load^10,11^. Biological processes, such as the circadian rhythms, mainly associated with sleep-wake cycles, are also thought to interact with time perception. In that phenomenon, sometimes called circadian timing, a coupling between circadian rhythms and a specific aspect of time perception occurs. An example of circadian timing would be a coupling between the time of day and the perceived duration of an event.

The dominant model of time perception in the literature proposes an ’internal clock’ in which an ’accumulator’ gathers the signals of a ’pacemaker’^1,12^ possibly oscillating at a fixed rate and which could be regulated by the circadian system (for review see^13^). Circadian rhythms follow a 24-hour cycle and respond primarily to light^14^ and the acetylcholine neurotransmitter tied to the parasympathetic nervous system important to sleep-wakefulness cycles^15^. Given this, manipulation of exposure to light is the most common experimental intervention used to study circadian timing. There’s substantial evidence suggesting that short-term interval timing, at a second to minutes range, displays circadian dependency on light and dark cycles both in human and other mammals^16–22^, although some studies put in question this relationship^23,24^. The key factor in this effect is thought to be retinal sensitivity to light and its retinohypothalamic projection onto the suprachiasmatic nucleus (SCN)—hypothesized synchronization hub of the mammalian circadian system^13,25^. The effect of light is thought to be independent of its source and the only relevant factor are its intensity and presence. The sun is the main natural source of light and its position is thought to be the main time-of-day cue, especially under equal intensity. Given this, it is natural to think that the sun gives the circadian system important cues and this is why it can be considered a zeitgeber (from German "time giver").

Considering that VR affords the creation of highly parameterizable environments, it gives us a distinct possibility to study environmental zeitgebers in relationship to time perception. Perhaps the most convincing evidence was provided by research on the use of VR in cancer treatment. Patients who spent their chemotherapy session in VR estimated the time of the session to be shorter^40^ and experienced less unpleasant symptoms related to chemotherapy. This is a clear example of the use of VR exposure to alter the experience of time. Furthermore, there is some evidence that mere replacement of the monitor with a VR headset compresses time^41^ and the design of the VR environment can affect time perception^42^ with more realistic and engaging designs. These findings triggered research that specializes in developing VR tools that allow for manipulation of the subjective experience of time^43,44^. The possibility to flexibly design the virtual environment facilitates testing hypothesis about the special coupling that is thought to exist between the circadian system and time perception. One study^28^ reported that the speed of the sun on the horizon affects the subjective experience of time in VR. This is a surprising and interesting finding given that not many other manipulations have been shown to exert significant effects, few of the possible zeitgebers and manipulations have been tested, and the studies that attempt to do this are mostly inconclusive. For example, Liao et al.^26^ tested the effect of visual (luminance) and auditory (ticking clock) zeitgebers on time perception in VR and report a weak interaction between cognitive load and auditory zeitgebers, which was reported as null in another study by Gutierrez et al.^27^. Furthermore, although color seems to have some effect on short-term interval timing in non-VR conditions, it was reported null on long intervals in VR^27^. The results that were reported significant were accompanied by very low effect sizes^26,28^.

To establish the robustness of the sun-speed effect reported by Schatzschneider et al.^28^, we attempted a conceptual replication^29^. We reproduced the virtual environment as close as possible to the original. To decrease the duration of the experiment we reduced the number of experimental conditions to those found significant in the original study and decreased the duration of each trial from 10 to 6 minutes. We also fully randomized experimental conditions to correct for the linearity of the paradigm. In this study, we will extend the hypothesis of the original study and apart from the original hypothesis on the effect of the movement of the sun, cognitive load, and immersion, we test the relationship between time perception and fatigue as well as aversive simulator symptoms introduced as confound control.

## Results

### Duration judgments

The sun was a constant component of the environment and was designed to move with either its natural speed on the horizon or to not move at all. Participants completed 8 trials in total, 6 minutes each, and were not informed of the fact that trials were of equal length, nor of the number of trials they would complete. Moreover, throughout the experiment participants listened to ambient sounds of sea waves and wind through headphones.

We experimentally manipulated the level of immersion by asking participants to complete the task in both a non-immersive and an immersive environment, in front of the LCD monitor or with the head mounted device (HMD) respectively. In order to probe the effect of cognitive load, as in the original study, in half of the trials participants were asked to complete the classical n-back task^30^. These experimental conditions amount to 2 (immersion) × 2 (sun movement) × 2 (cognitive load) design. At the end of each trial, participants were asked to estimate the duration of the time of the passed trial in seconds, following Schatzschneider et al. 2016 design. At the beginning and the end of the experimental session participants were also asked to complete a Simulator Sickness Questionnaire (SSQ), the change of this score is used as Simulator Sickness Score. To test the effects of each experimental condition we run a 2 × 2 × 2 Repeated measures ANOVA, with the Simulator Sickness Score as a covariate in a separate run. Results were adjusted for multiple comparison with a Bonferroni correction where needed.

#### Sun

**Figure 1:**
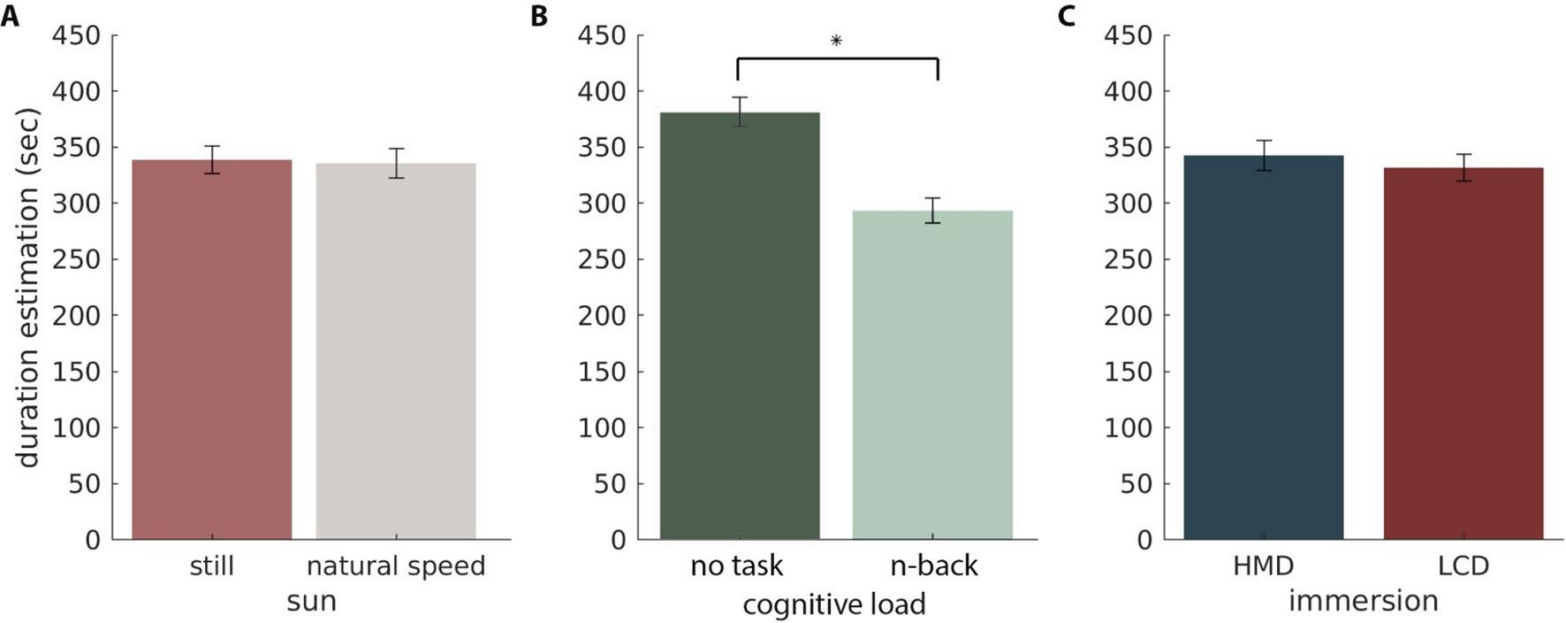
Mean duration estimation for each experimental condition. Error bars indicate SEM. We found no significant effect of sun (**A**) nor immersion (**C**). We found a significant effect of cognitive load (**B**). Post-hoc tests indicated that subjects estimated time to be shorter in the condition with an n-back task compared to the condition with no task (*t*(36) = 8*.*307*, p* = 6*.*876*e* − 10*,d* = 0*.*59).

**Figure 2:**
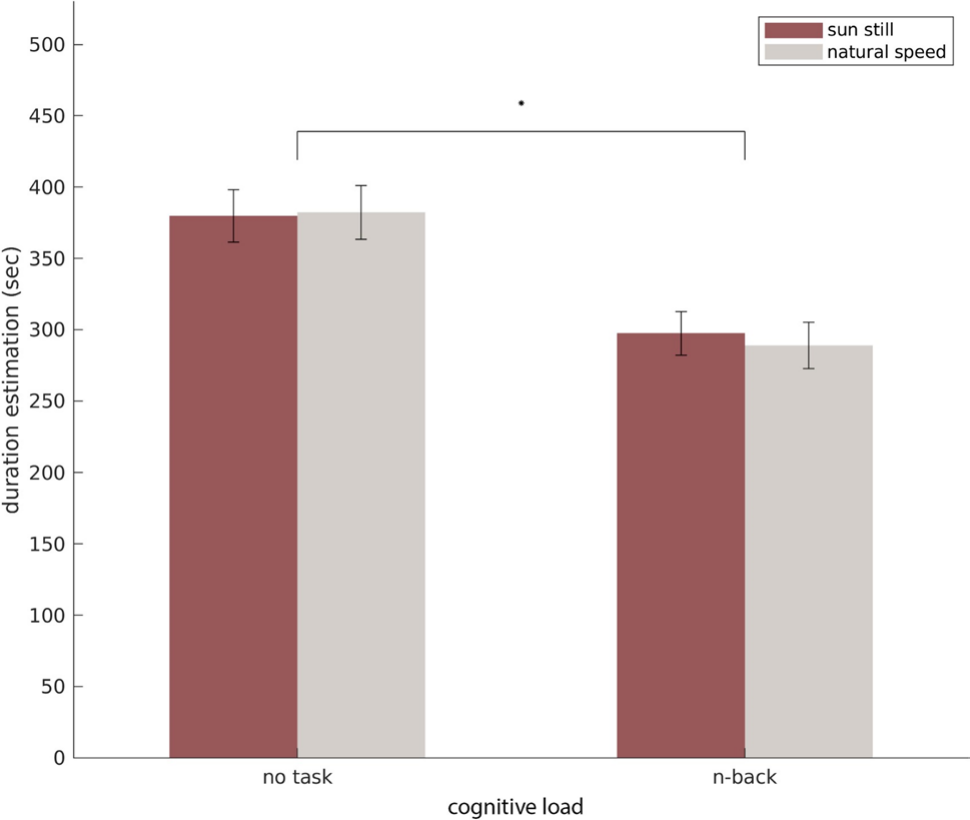
Mean estimation as an interaction between the sun and cognitive load. We did not replicate the effect from the original study.

First, we tested the effect of the manipulation of the sun. In the original study authors found that when no task was executed, participants estimated the time to be longer when the sun was still than when it moved at its natural speed. We found no significant effect of the manipulation of the speed of the movement of the sun on time estimation (F(1,36) = 0.103, *p* = 0.750, Fig. 1A) even when we look only at the condition with no-task (two-tailed *t*(36) = − 0*.*191*, p* = 1, adjusted for multiple comparisons, Fig. 2). To make sure that the effect was not inhibited by the unpleasant simulator effect we included the score as a covariate to the ANOVA analysis. Although adding the SSQ score improves fit of the model, (residuals(full + SSQ) < residuals (full), where residual = data − fit), it has no significant effect on the sun, immersion, or interaction between sun and immersion.

#### Cognitive load

We replicated the classical cognitive load result^11^ and found a significant effect of the task on trial duration estimation (*F*(1*,*36) = 69.013; *p* = 6.874e-10, *η*_*p*_^2^= 0.657). Post-hoc test revealed duration estimation to be shorter when participants performed a task than when they were passively present in the environment (*t*(36) = 8*.*307, *p* = 6*.*876*e*−10*,d* = 0*.*59, Fig. 1B).

#### Immersion

**Figure 3:**
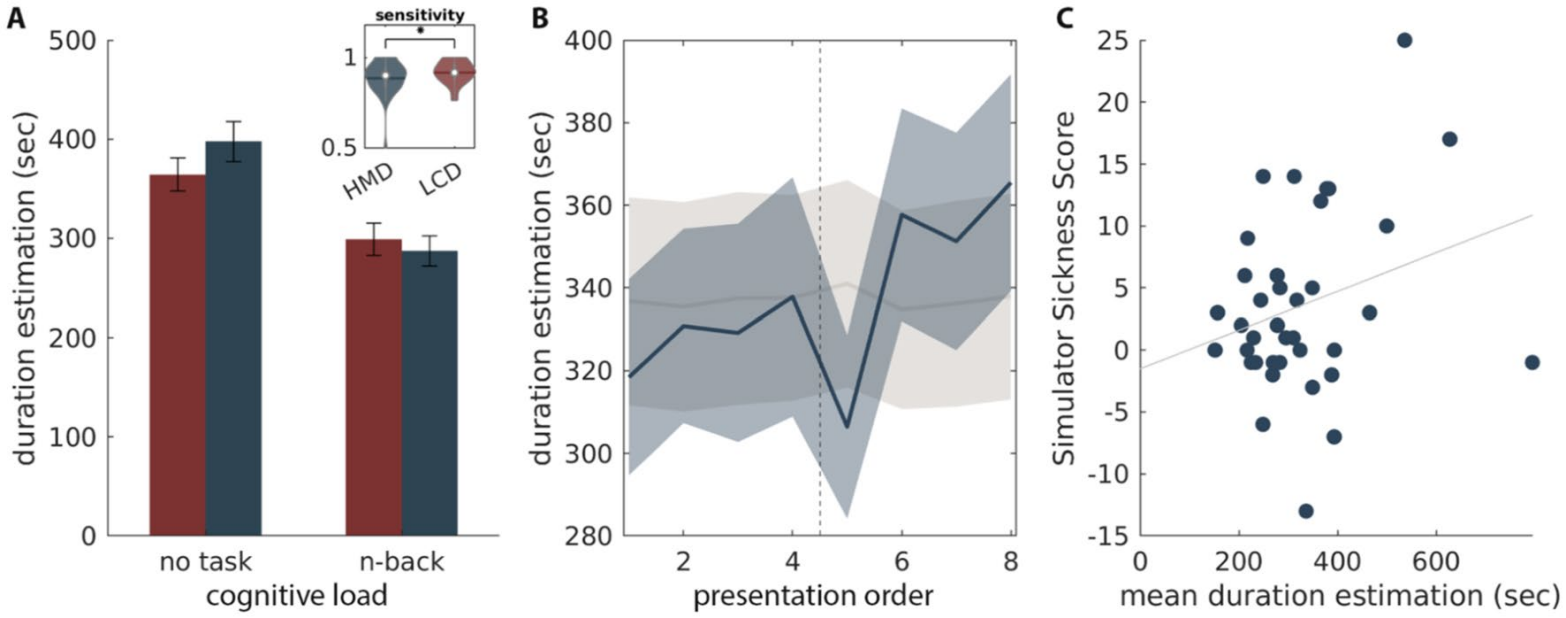
(**A**) Mean estimation as interaction between immersion and cognitive load. *Inset* Performance (sensitivity index A’) during the n-back task for the two immersion levels. (**B**) Average time estimation over the course of the experiment, regardless the experimental condition. In gray label shuffled permutation, shaded area represents standard deviation. (**C**) Scatter plot of the mean duration estimation during HMD trials and the SSQ score. Each dot represents one participant.

Schatzschneider et al.^28^ reported shorter duration estimation in non-immersive (LCD) than in immersive (HMD) environments. We did not replicate this result and no effect of the environment was found on duration estimation (*F*(1*,*36) = 0*.*588; *p* = 0*.*448, Fig. 1C), however, we found a significant interaction between cognitive load and immersion (*F*(1*,*36) = 6*.*854; *p* = 0*.*013, $\upeta _{P}^{2} = 0.16$)—when no task was assigned participants estimated time to be longer in an immersive environment, but when participants performed a task, they estimated the duration to be longer in a non-immersive environment (Fig. 3A). We initially thought that this is again a cognitive load effect—in immersive environment participants were exposed to fewer distractors and could focus on the task more, and engage more cognitive resources. However, performance was slightly higher in the LCD condition (paired *t*(73) = 2*.*7156, *p* = 0*.*0083, Fig. 3A *inset*). In light of these results, we hypothesize that HMD was somehow a more challenging environment. In the non-immersive environment, in the condition with no task, participants were exposed to the rest of the experimental room, which resulted in a more complex environment as opposed to the HMD condition where only the virtual island could be observed.

#### Simulator Sickness symptoms—physiological arousal

Although negative simulator symptoms did not specifically affect the effect of the sun on time estimation, we tested whether it had any effect on average time estimation. To assess the statistical significance of this relationship we regressed the SSQ score on duration estimation measurements. The SSQ score explained a significant amount of the variance of the duration estimation (*F*(1*,*294) = 9*.*590*, p* = 0*.*002*,R* = 0*.*178*,R*^2^ = 0*.*032). The regression coefficient (*B* = 3*.*765*,*95%*,CI* = [1*.*372*,*6*.*156]) indicated that an increase in SSQ score by 1 increased, on average, the duration estimation by 4 seconds (Fig. 3C). We hypothesize that this effect of aversive simulator sickness symptoms, like nausea or vertigo, is mediated by the link between physiological arousal and time perception. It has previously been shown that various types of arousal affect interval timing^4–6^, especially unpleasant stimuli dilate time perception^31^. Although Schatzschneider et al.^28^ also collected SSQ responses, they did not provide analysis beyond score change.

#### Fatigue

As stated above, we did not replicate the effect of immersion on time duration estimation. Although the effect reported in the original paper was not significant, it did appear as a strong trend, at least for conditions with a task. In the original paradigm, participants had always started with a non-immersive block and continued onto an immersive block. Such a linear design is confounded by fatigue. At the beginning of the experiment, participants may estimate the duration to be shorter just because they are less tired than at the end, so we hypothesize that this is the reason we did not replicate the immersion effect reported by Schatzschneider et al.^28^. In our design, we controlled for this effect by randomizing the immersion blocks. Whatever the condition with which participants have started or finished the experiment, they estimated the time to be shorter at the beginning of the experiment than at the end (Fig. 3B) with a transient constricting effect of the environment change mid-experiment.

### Bayesian analysis

**Figure 4:**
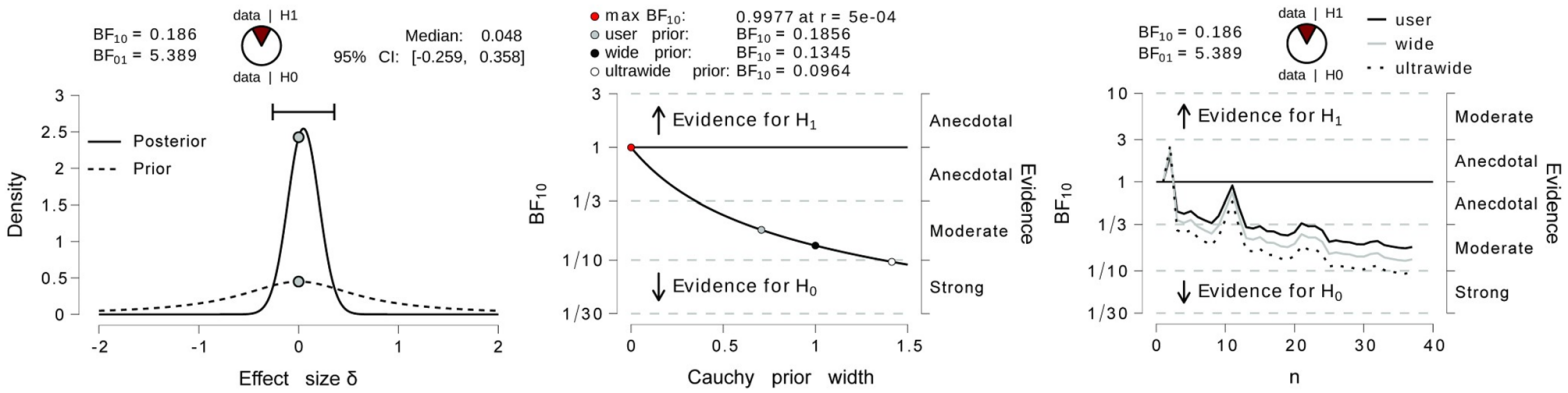
Results of the post-hoc Bayesian paired sample t-test between the two levels of sun manipulation. On the left, the effect size as a function of the prior and posterior density. In the middle *BF*_10_ as a function of tested prior. On the right, accumulation of evidence towards H0 as a function of the number of samples (participants).

Non-significant results of frequentist tests do not discriminate between “absence of evidence” and “evidence of absence”. To test our ability to present evidence in favor of the null hypothesis (no effects of sun movement on time perception) we went beyond the frequentist approach, turned to Bayesian Inference, and conducted Bayesian Repeated ANOVA and separate Bayesian Paired-Sample t-tests for each condition. We first conducted a Bayesian Repeated Measures ANOVA on the data with the experimental conditions [immersion (2) × task (2) sun (2)] as within-subjects factors. We used the default prior options for the effects (i.e., *r* = 0*.*5 for the fixed effects). To assess the robustness of the result, we also repeat the analysis for two different prior specifications (details in the ''Methods'' section). Analysis of effects indicates moderate to strong evidence for exclusion of immersion (*BF*_*excl*_ = 4*.*803, where *BF*_*excl*_ is the change from prior to posterior exclusion odds for model-averaged results, our notation follows JASP manual), sun (*BF*_*excl*_ = 16*.*815), and all interactions between the two. In fact, only the model with a single cognitive load term had *BF*_*excl*_ smaller than 1 (*BF*_*excl*_ = 3*.*724*e*−13). Post-hoc tests indicated robustness of these findings to prior width (Figs. 4, S2). We can therefore conclude that in our data we can observe moderately strong evidence for no effect of the manipulation of sun or immersion on duration estimation.

## Discussion

We found that the movement of the sun on the horizon did not affect interval timing in VR. Although the hypothesis was compelling, we found no evidence of coupling between the movement of the sun and time perception. We speculate that the inability to replicate the sun-speed manipulation effect is at least in part the result of stringent confound control, which was not in place in the Schatzschneider et al. study. To assess the strength of evidence in favor of the null hypothesis we carried out a post-hoc Bayesian Inference analysis to estimate the evidence for a lack of relationship between sun movement and duration perception. Given this choice of analysis, our negative results are a step towards uncovering a relationship between the environment, the circadian system, and human time perception. Instead of the hypothesized effect of the sun. We presented results suggesting that fatigue and aversive symptoms of the simulator like nausea and vertigo do affect interval timing, and therefore should be given careful consideration during experimental design. We reported the classical cognitive load effect and finally used Bayesian inference to provide evidence for the lack of effect of the sun on duration perception. There are several potential reasons why the replication had failed, among them the adjusted paradigm and larger sample size. Important limitation of our replication lays in reduction of trial duration from 10 minutes to 6 minutes. One could argue that the effect of the sun required intervals longer than 6 minutes, i.e. 10 minutes like in the original study. To address this issue further investigations are needed, however, we argue that according to the scalar property of interval timing the relative precision of estimation should decrease along with the length of period to estimate^32^. Thus, the variability of the response to the sun should decrease with shorter durations, at least at minutes range, and be more pronounced at longer intervals. It is worth noting that the majority of research that concerns that model applies to shorter durations, milliseconds to seconds range, and some studies question the existence of scalar property of time perception^33,34^, or even show opposite results^35^. Regardless of the direction of the relationship, the relative precision of estimation changes along the length of the period to estimate, and therefore our reduced trial interval sets a limitation on the conclusions we can draw from our results. It is also a possibility that for the effect of sun manipulation requires longer exposure i.e. the size of the effect of the sun increases with exposure time. Nevertheless, the confounds of the original experiment are a possible alternative reason for our difficulty to replicate the effect in question , for example, the fact that participants were allowed to take a rest by closing their eyes but not taking the HMD off as pointed out by Gutierrez^27^. Future research should aim to address the question of the relationship between the interval length of the exposure to manipulated sun and subjective duration estimation. Furthermore, if the effect of sun movement depends on the interval of exposure, it should be questioned whether zeitgebers tested before, like clock ticking, also don’t show such dependence.

In conclusion, although VR has been shown to be an interesting tool to manipulate human time perception, so far no given manipulations of the environmental zeitgebers were proven to affect human time perception in VR beyond manipulations proven to work outside of VR as well. What we described instead is another possibility for circadian timing coupling between the state of the cholinergic nervous system (fatigue, nausea, and simulator sickness) and time perception^36^. This may be a good direction for future studies that aim to find a connection between these two important components of timing processes in the human brain.

## Methods

All methods and procedures used in this study conformed to ethical guidelines for testing human participants (WMA Declaration of Helsinki) and were approved by the Institute of Philosophy of the Jagiellonian University evaluation committee. All the participants gave written informed consent before the experiment.

### Participants

Power analysis indicated a total sample size for *η*_*p*_^2^ = 0.06^28^ and correlation among repeated measure equal to 0.2 is 36 participants. We, therefore, recruited 38 human participants (24 females, 4 preferred not to answer, age M = 21.7; SD = 2.46) for the experiment. We excluded 1 participant—due to malfunction of the acquisition system, one of the experimental conditions was collected twice. The final sample size was therefore N=37. Healthy participants with no history of psychological or neurological disorders with normal or corrected-to-normal vision were recruited through the popular social media platform. Importantly, the correlation among repeated measures in our experiment amounted to *r* ∼ 0.6 which resulted in a sufficient sample size of 19 participants.

### Procedure

The 3D virtual environment of a small uninhabited island was designed using Unity 5.6.4f1 (see Figure S1, left panel) and the implementation of sun movement was realized using the Time of Day System Free 1.2.8 asset. The experiment was carried out using Oculus DK2 (the same model was used in the original study), on a computer able to maintain 75 frames per second in the environment designed for the experiment (as this version of Oculus has 75Hz refresh rate), equipped with Nvidia GeForce GTX 1060 graphics card, 16GB RAM and AMD Ryzen 5 1500X processor. The project has also been tested using newer equipment and works with Oculus Quest 2 connected to a PC via Air Link. The goal was to create an environment that would be most similar to the one used in the original study. Identically to the original experiment, the virtual time of the experiment was set to morning, soon after sunrise, and the position of the sun was horizontally randomized (± 40 degrees from the center of the field view) for every scene load (every variant), to ensure that the participants did not always have the sun directly in front of them. Participants were invited to the experiment at various times of the day and we found no effect of testing time and mean duration estimation (r = 0.016, *p*=0.924). We introduced sound to the experiment—in all variants the participants had headphones on with quiet ambient sounds of sea waves and wind being played to increase immersion level. The HMD allowed the participants to freely look around, but they were instructed to look ahead as they were seated, and the 3D model of the chair used in the experiment corresponded to the standard office chair that was used in the laboratory room. All the instructions appeared as slides within the software to ensure that there was no need to take off the HMD between trials.

### Paradigm

The experimental conditions amount to 2 (immersion) × 2 (sun movement) × 2 (cognitive load) design. The two levels of immersion were defined by the experimental setup—participants either completed the task with a head-mounted device or on the LCD screen. The two levels of the sun manipulation condition were defined by the speed of the sun on the horizon. Levels of cognitive load were binary—participants either completed an n-back task or were passively present in the environment. To control for fatigue, we randomized the immersion blocks. All other conditions (task and sun) were randomized within blocks. To decrease the duration of the experiment we decided to decrease the number of conditions from the original paradigm design—2 (immersion) × 3 (cognitive load) × 3 (sun speed). The cognitive load levels included no task, n-back task, and spatial orientation task. Since there was no significant difference in interval estimation between spatial and n-back conditions we decided to use only one. The levels of the sun speed gain—no movement, natural speed, and amplified speed—were deprecated to two levels: no movement and natural speed, as analogically there was no significant difference in interval estimation between natural and amplified sun speed in the original experiment. In the present study, a no sun movement condition was introduced to the LCD variant to counterbalance the design (in the original study only one—natural speed condition—was used).

Participants began the experiment by completing the Simulator Sickness Questionnaire. Next they did a short (couple of minutes) training with the HMD. After the participants indicated they understood the procedure, they started the main part of the experiment. After completing each of the 8 trials in two blocks participants were asked to estimate the passed interval by changing the value in minutes using arrows on the gamepad (one quick tap => one second change in value, continuous press => fast change of value), and then accepted the value using the “X” button. After completion of all trials in two blocks participants were asked to fill out the the SSQ again. The change in the Simulator Sickness score (calculated as in the original paper^28^) was used in the analysis as indicator of the aversive simulator symptoms. The total procedure lasted about an hour.

#### n-back task

We used the 2-back version classical n-back task (Figure S1, right panel). Participants were instructed to determine if the presently displayed letter is the same as the one that was displayed two letters before (example: A-B-A -> TRUE, B-C-A -> FALSE). Each letter was displayed for 500ms. The inter stimuli interval was randomized between each letter and drawn from a uniform distribution between 1.1 and 1.5 seconds. Participants indicated letter matching by button press.

### Data analysis

All figures (except the results of Bayesian analysis, which were generated in JASP) were generated with MATLAB R2021a (Copyright 1984-2022 The MathWorks, Inc.). RM ANOVA, Bayesian RM ANOVA, and all post-hot tests were done in JASP^37^ (0.16.1.). Performance during the task was calculated as the sensitivity index *A*′—version of the *d*′ index for unequal variances between false alarms and hit rates^38^) in MATLAB. Results in the text are reported as mean ± standard error of the mean (SEM). Repeated measures ANOVA with 2×2×2 design was computed with JASP functionality. Post-hoc tests were run with Bonferroni correction for multiple comparisons. In a separate run, we added a change in the SSQ score (indicated as diffSSQ in the JASP report) as a covariate. To estimate the strength of evidence for H0 or H1 for each RM factor (sun, immersion, and cognitive load) with run Bayesian Repeated measure ANOVA (also in JASP) with three prior *r* scale values 0.25,0.5 and 0.75—the conclusions were not affected by the prior value. Post-hoc averaged group-level Bayesian t-tests were conducted for each RM factor. Robustness analysis was conducted to check for the robustness of the results to Cauchy’s prior width. All reported *p* values are two-tailed.

## Supplementary Information

Supplementary Information.

## Data Availability

All data used in the analysis and JASP reports, as well as Unity code used to render the virtual environment and execute the experiment, are available online on Open Science Framework.
