# Supplementary material for: Cognitive load, fatigue and aversive simulator symptoms but not manipulated zeitgebers affect duration perception in virtual reality
Source: Sci Rep. 2022 Sep 20;12:15689. doi: 10.1038/s41598-022-18520-1 (PMC9489727; doi:10.1038/s41598-022-18520-1)
Supplement: Supplementary file 1 — Supplementary Information. [file 41598_2022_18520_MOESM1_ESM.docx]

# Supplementary materials


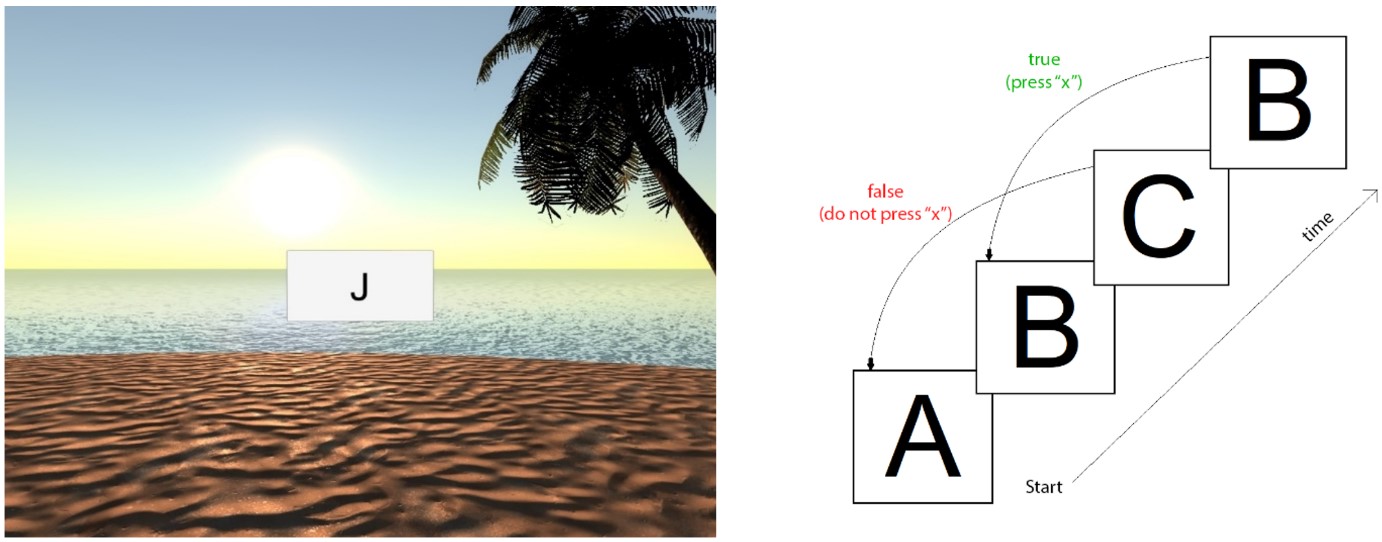


**Figure S1.** On the left the island environment and an example of the trial of the n-back task. On the right is the rationale of the two-back letter verbal task.


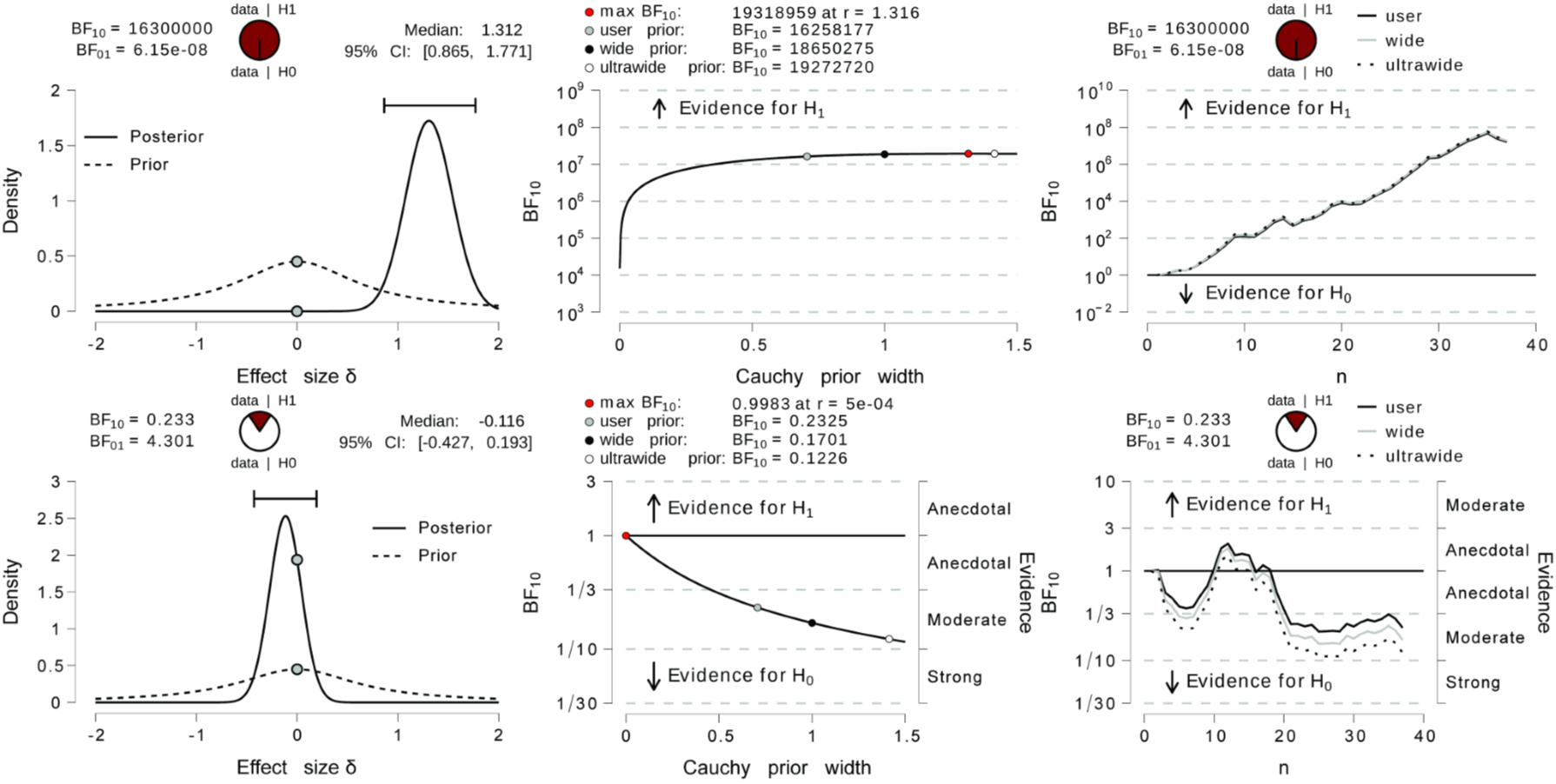


**Figure S2.** Results of the post-hoc bayesian paired sample t-test between the two levels of cognitive load manipulation ( top ) and immersion (bottom). On the left, the effect size as a function of the prior and posterior density. In the middle *BF*_10_ as a function of chosen prior. On the right accumulated evidence as a function of a number of samples ( participants ).
